# Supplementary material for: Characterization, Selection, and Trans-Species Polymorphism in the MHC Class II of Heermann’s Gull (Charadriiformes)
Source: Genes (Basel). 2022 May 20;13(5):917. doi: 10.3390/genes13050917 (PMC9140796; doi:10.3390/genes13050917)
Supplement: Supplementary file 1 [file genes-13-00917-s001.zip › Figures S1 and S2, Tables S1 and S2.pdf]

genes

MDPI

Supplementary

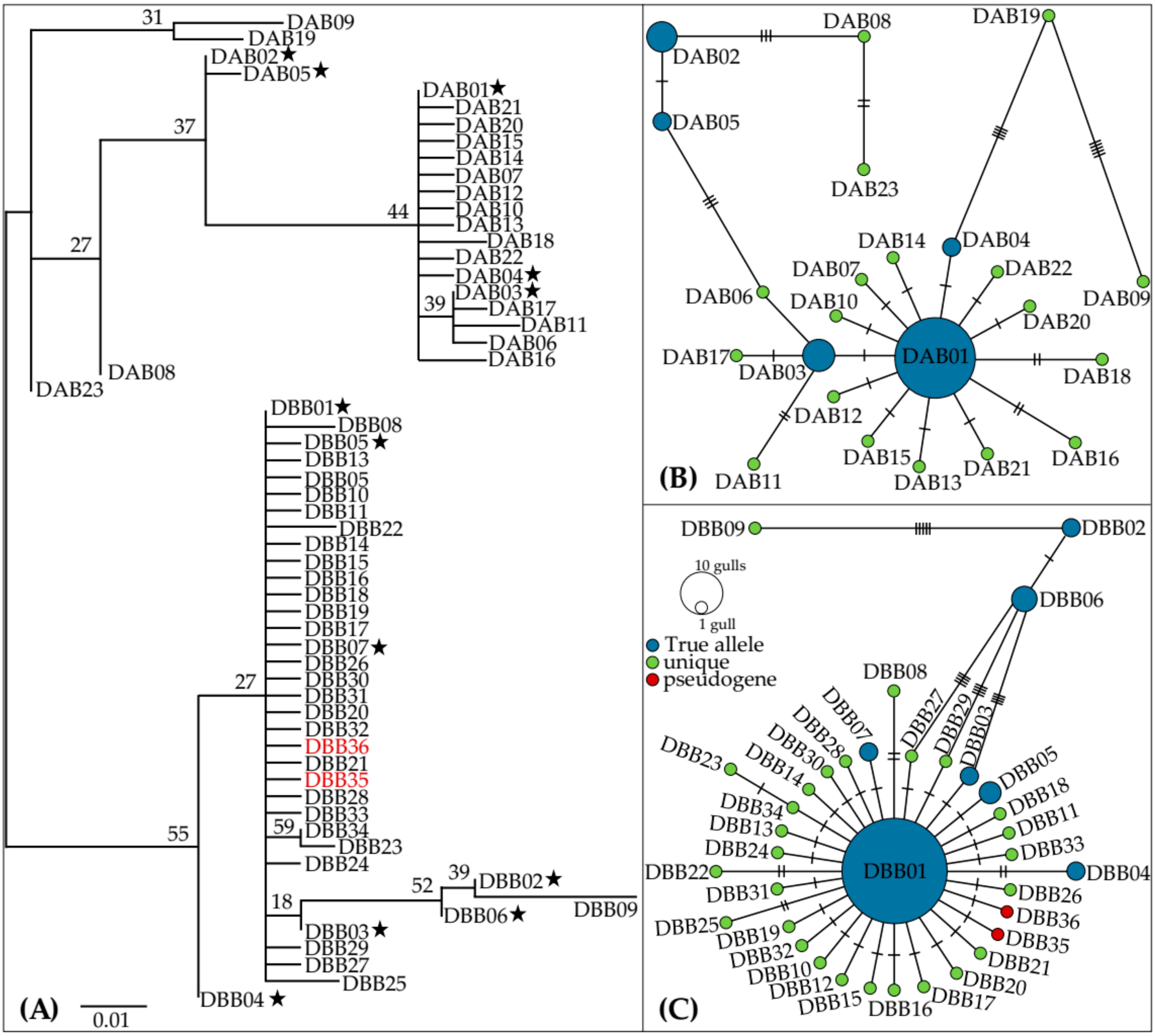

Figure S1. (**A**) Maximum likelihood tree of fifty-nine MHCIIB haplotypes suggesting at least two loci in Heermann's Gull (LogL = -838.02). Putative true alleles are highlighted with dark stars, the remaining are unique alleles. Within these, only two showed stop codons (in red). The tree was obtained using the GTR+I+G model and 1,000 bootstraps. Branch lengths correspond to the number of substitutions per site. The percentage of trees in which the associated alleles clustered together is shown next to the branches. (**B**) Twenty-three alleles of the DAB locus were found but only five were considered as true (found in two or more individuals). (**C**) Thirty-six alleles of the locus DBB were found showing seven true alleles, twenty-seven unique alleles, and two pseudogenes.

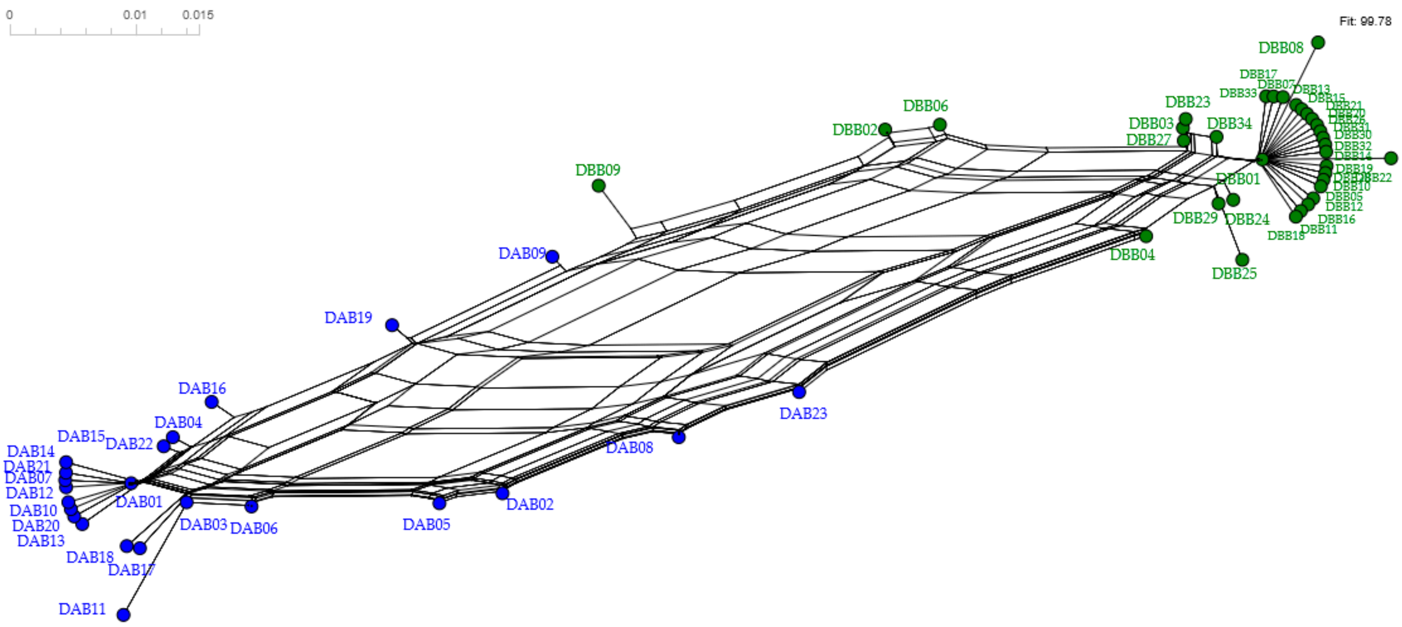

Figure S2. Neighbor-net for all the MHCIIB alleles recovered in Heermann's Gull.

Table S1. Structure assessment for the most common alleles (Lahe-DAB*01 and LaheDBB*01) found in Heermann's Gull. Chicken (6kvm) and humans (4h25) templates were used to assess each model in both alleles with SWISS-MODEL [65].

**Assessment**	**Lahe-DAB*01**	**Lahe-DAB*01**	**Lahe-DBB*01**	**Lahe-DBB*01**	
Template	6kvm	4h25	6kvm	4h25	**Ideal Case**
% Identity	60.61%	59.09%	59.09%	56.06%	as high as possible (above 40%)
GMQE	0.85	0.86	0.85	0.84	1
QMEAN DisCo Global	0.81±0.11	0.78±0.11	0.80±0.11	0.79±0.11	1±0.00
QMEAN Z-score	-0.92	-0.48	-1.44	-1.32	zero (below -4.0 indicates model with low quality)
MolProbity	0.50	0.69	0.50	1.45	as low as possible
Ramachandran favored	98.44%	96.88%	98.44%	98.44%	> 98%
Bad angles	4 / 762	11 / 762	3 / 750	5 / 750	zero
C-Beta Deviations	1 (B15, ASN)	0	1 (B15, ASN)	0	zero
Rotamer outliers	0	0	0	1.82% (B17, GLU)	< 1%

Table S2. Analysis of selection in Heermann's Gull using PBR sites inferred from humans. Neutral (Ha: *dN=dS*), positive (Ha: *dN>dS*), and purifying (Ha: *dN<dS*) evolution statistics for z- tests are shown with their respective probability values among parentheses.

Species	Domain	*dN*	*dS*	*dN-dS*	*ω*	*dN = dS*	*dN > dS*	*dN < dS*
Larus heermanni (n = 57)	All	0.07 ± 0.02	0.02 ± 0.01	0.05 ± 0.02	3.18	2.43 (0.02)	2.46 (0.01)	-2.33 (1)
	PBR	0.15 ± 0.06	0.04 ± 0.03	0.11 ± 0.07	3.12	1.56 (0.12)	1.53 (0.06)	-1.66 (1)
	Non PBR	0.04 ± 0.01	0.02 ± 0.01	0.03 ± 0.01	3.20	1.82 (0.07)	1.77 (0.04)	-1.85 (1)
DAB (n = 23)	All	0.03 ± 0.01	0.01 ± 0.01	0.01 ± 0.01	3.27	2.20 (0.03)	2.04 (0.02)	-2.01 (1)
	PBR	0.06 ± 0.02	0.01 ± 0.01	0.04 ± 0.02	3.64	2.01 (0.05)	1.98 (0.02)	-2.09 (1)
	non PBR	0.02 ± 0.01	0.01 ± 0.01	0.01 ± 0.01	3.15	1.00 (0.32)	1.00 (0.16)	-0.93 (1)
DBB (n = 34)	All	0.02 ± 0.00	0.01 ± 0.00	0.01 ± 0.00	3.12	2.45 (0.02)	2.43 (0.01)	-2.52 (1)
	PBR	0.03 ± 0.01	0.00 ± 0.00	0.03 ± 0.01	2.84	2.50 (0.01)	2.33 (0.01)	-2.37 (1)
	Non PBR	0.01 ± 0.00	0.01 ± 0.01	0.01 ± 0.00	3.24	1.21 (0.23)	1.18 (0.12)	-1.22 (1)
